# Dangers of Colds

**Published:** 1899-12

**Authors:** 


### Dangers of Colds.

In the October number of the *Medical Brief* we find some hints valuable to the profession at this time of the year:

The greatly increased sensitiveness of the individual to atmospheric changes, resulting from the artificial conditions of life incident to civilization, makes catarrh of some organ or organs the pathological basis of most diseases prevailing in this climate.

Catarrhs begin as colds, which, uncured, or following one another in rapid sequence, produce a state of lowered vitality, so that the patient does not recuperate and get entirely well after each fresh attack of cold, although the acute stage passes off. Gradually the chronic cold, or catarrh, extends from one organ or part to another by contiguity, until the whole system is diseased and torpid, and the work of regeneration becomes such a herculean task, few patients are willing to keep at it, to faithfully and systematically carry out the doctor's instructions during the long, weary time essential to effect a radical cure.

Now is the time when the seeds of catarrhs are being sown freely in the shape of autumn cold. A cold does not always, or necessarily, settle in the nose or throat. It may select the stomach, bowels, liver, kidney or other organ, as the point of attack. If not promptly treated and cured, the majority of patients will remain "under the weather," and the disease, like a smouldering fire, will gradually spread and involve adjacent parts.

The symptoms of a cold, or catarrh, after the acute stage has passed, do not disable the patient to any marked extent, unfortunately. He feels miserable and dull, but is able to keep going, and to perform his tasks in a leaden sort of way, and so he pays but little attention to his condition, accepts the burden of impaired health with more or less resignation, occasionally making short, spasmodic efforts to recover lost ground, but never setting about the work of regeneration in a scientific manner, with the aid and constant supervision of a competent and sympathetic physician.

If the profession and public would only realize that the quiet, insidious sapping of vital forces due to chronic catarrh of various organs is more dangerous and far-reaching in its effects than the most severe case of pneumonia or diphtheria, intelligently treated, the majority of people would enjoy good health, and the medical profession a better reputation for usefulness; whereas now, out of a crowd of fifty men and women, it would be difficult to pick out half a dozen in perfect health.

Teach your patients that a cold is a very serious thing, and that any disturbance of the health resulting in stoppage of the secretions, shivering sensations, a dry, hot skin and congestion of internal organs is a cold, whether it is termed technically rhinitis, bronchitis, gastritis, enteritis, hepatitis, nephritis, cystitis or any other itis. Show them the importance of curing it at once before other organs are involved; before the vital forces of the system are depressed, and the nerves and circulation of the diseased part become permanently altered by habitual morbid action.

